# The Effects of Early Postnatal Alcohol Exposure on Bone Molecular Composition in a Mouse Model

**DOI:** 10.1111/acer.70331

**Published:** 2026-06-05

**Authors:** Ilknur Dursun, Birsen Elibol, Sebnem Garip Ustaoglu

**Affiliations:** ^1^ Department of Physiology, Faculty of Medicine Istanbul Health and Technology University Istanbul Turkiye; ^2^ Department of Medical Biology, Faculty of Medicine Istanbul Medeniyet University Istanbul Turkiye; ^3^ Department of Biochemistry, Faculty of Pharmacy Istanbul Aydin University Istanbul Turkiye; ^4^ Department of Medical Biochemistry, Faculty of Medicine Altinbas University Istanbul Turkiye

**Keywords:** ATR‐FTIR spectroscopy, bone, bone metabolism, bone quality spectral markers, bone‐related hormonal markers, gavage‐related stress, postnatal alcohol exposure

## Abstract

**Background:**

Perinatal exposure to alcohol is a critical risk factor for long‐term skeletal health in humans and has been demonstrated in rodent models. Early postnatal alcohol gavage exposure in mice is used to study third trimester toxicity in humans. However, further research is required to fully characterize the effects of alcohol on bone molecular composition and distinguish them from the alterations associated with gavage‐related stress in mouse models.

**Methods:**

Female mouse pups were assigned to untreated control (C), gavage control (GC), and alcohol‐treated (A) groups. Between postnatal days 3 and 20, the A group received ethanol (3.0 g/kg/day) by intragastric gavage, while the GC group underwent the same gavage procedure without any solution (neither milk nor ethanol). At postnatal day 90, femoral bone molecular composition and bone metabolism were evaluated by attenuated total reflection Fourier‐transform infrared (ATR‐FTIR) spectroscopy and biochemical studies.

**Results:**

Several ATR‐FTIR‐derived matrix and mineral‐related alterations, including reduced total protein‐related indices, lower collagen cross‐link ratio, reduced mineral‐to‐matrix ratio, and increased crystallinity, were observed in both GC and A groups relative to untreated controls, indicating a substantial long‐term influence of the gavage procedure itself. Compared with the GC, the A group showed additional selective differences, most notably reduced relative carbonate content and increased CTX‐I levels. In the C group, serum 25(OH)D, PTH, and calcitonin levels differed from both GC and A groups, whereas osteocalcin was increased in the GC group relative to the C group, and no significant difference was observed between the GC and A groups.

**Conclusion:**

Prolonged neonatal gavage was associated with substantial long‐term changes in bone molecular composition and several circulating bone‐related hormonal markers. Against this background, postnatal alcohol exposure was associated with additional selective differences, particularly in CTX‐I and relative carbonate content. These findings highlight the importance of discriminating between the effect of the procedure and the effect of alcohol in developmental models based on repeated neonatal gavage.

## Graphical abstract

Early postnatal alcohol exposure was associated with long‐term changes in adult bone molecular composition and increased CTX‐I, indicating persistent effects on skeletal biology. Overlap with gavage‐related changes highlights the need to distinguish alcohol‐specific effects from procedural influences.

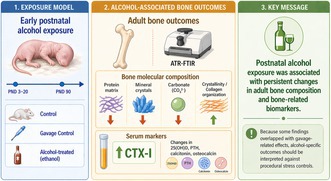

## 1 Introduction

Developmental alcohol exposure has been reported to affect most of the vital tissues, a result of which may cause permanent injuries in the body. In our previous studies, we also have demonstrated the adverse effects of alcohol exposure during the critical developmental period on different tissues (Algburi et al. 2022; Dursun et al. 2011). The skeletal system is one of the systems most vulnerable to damage in this stage of development, as the mineralization and maturation of the extracellular matrix are highly intensive processes. Accordingly, it was reported that developmental alcohol exposure negatively impacts skeletal development and has long‐lasting effects on skeletal health. Both clinically and experimentally, perinatal alcohol exposure has been shown to reduce bone density and alter the morphology of the different regions of bone, resulting in an increased potential for fracture (Ertem et al. 2006; Pillay et al. 2024; Simpson et al. 2005; Young et al. 2022). Moreover, alcohol exposure has been shown to exert a direct cytotoxic effect on cells as well as to compromise vital hormonal regulatory mechanisms. Alcohol‐induced hypothalamic–pituitary–adrenal (HPA) axis disruption leads to calcium homeostasis impairment besides vitamin D deficiency and parathyroid hormone (PTH) suppression due to chronic alcohol exposure. All those hormonal alterations disturb bone turnover and skeletal integrity, resulting in lifelong implications on bone health (Moon et al. 2023; Wieczorek et al. 2015; Woolford et al. 2019).

Early postnatal alcohol exposure in rodent models is a critical tool for investigating the impacts of ethanol during a period that corresponds to the human third trimester (Patten et al. 2014). This stage is characterized by rapid tissue maturation and intensive mineralization, making the skeletal system particularly vulnerable to developmental toxicity. Consequently, this model is highly relevant for understanding the mechanical and molecular bone deficits observed in fetal alcohol spectrum disorders (FASD) resulting from heavy maternal alcohol consumption.

Intragastric gavage is a commonly used technique to obtain alcohol rodent models in experimental studies. However, this experimental technique can induce stress‐related physiological effects on the animals. One of those physiological effects includes the stress‐induced excess secretion of glucocorticoids, which in turn suppress osteogenesis and bone structure development (Suarez‐Bregua et al. 2018; Zhang et al. 2025). Thus, it is crucial to differentiate between the effects of alcohol toxicity and those of the gavage‐related stress when evaluating skeletal deficits. The inclusion of both intragastric gavage control and non‐treated control groups in the current study enables a clearer distinction between alcohol‐related and gavage‐related effects.

The present study assessed how early postnatal alcohol exposure and prolonged gavage influence bone molecular structure and bone‐related biochemical markers, with particular attention to distinguish alcohol‐specific effects from procedural effects, using attenuated total reflection Fourier‐transform infrared (ATR‐FTIR) spectroscopy and biochemical assays. The present study differs from earlier studies by combining molecular, structural, and biochemical investigations to provide a comprehensive characterization of the effects of postnatal alcohol exposure on bone health. This integrated approach provides a comprehensive evaluation of bone tissue quality. The mineral content, crystallinity, relative carbonate substitution, and collagen cross‐linking of the bone tissue were measured in order to provide an accurate reflection of the composition and mineral integrity of bone tissue. The findings reported herein provide novel insights by demonstrating a correlation between molecular alterations and functional biological impacts within a developmental frame.

## 2 Materials and Methods

### 2.1 Animals and Experimental Procedures

The adult C57BL/6 mice, including males (*n* = 5) and females (*n* = 7), were housed in a temperature‐ and humidity‐controlled environment (21°C ± 2°C and 62% ± 7% humidity) under a 12‐h light/dark cycle in Plexiglas cages. The mice had free access to standard chow and water. All procedures were conducted in accordance with the NIH Guide for the Care and Use of Laboratory Animals and the Declaration of Helsinki and were approved by the Bezmialem Vakif University Scientific Ethics Committee (Istanbul, Turkey; protocol 2017/99‐1). Animal studies and experimental procedure were carried out according to the ARRIVE guidelines. For breeding, a male was randomly selected and placed in a female's cage overnight; this was repeated nightly until a vaginal plug was observed. The day the plug was detected was considered confirmation of fertilization and designated gestational day 0 (GD0).

The day of birth was assigned as postnatal day 0 (PND 0). Female pups were then randomly assigned to three groups: control (C, *n* = 6), intragastric gavage control (GC, *n* = 6), and alcohol‐treated (A, *n* = 6). Between PND 3 and PND 20, the pups received 3.0 g/kg of ethanol per day in two equal intragastric doses, 2 h apart. Each dose was 0.0278 mL per gram of body weight. The administration of 3.0 g/kg/day of ethanol utilized in this study has been demonstrated in our prior research to consistently produce binge‐like blood alcohol concentrations. In strict adherence to ethical principles regarding the minimization of animal subjects, BAC measurements were not repeated here (Dursun et al. 2011). The GC group was intubated at the same frequency and timing but received neither ethanol nor milk. This approach was chosen because neonatal pups do not effectively regulate food intake; providing supplemental milk to gavage control groups has been shown to result in abnormal weight gain relative to non‐treated controls, as previously established (Kelly and Lawrence 2008).

On PDN 90 days, mice were anesthetized with intraperitoneal injection of ketamine–xylazine (ketamine 80–100 mg/kg IP and xylazine 10–12.5 mg/kg IP) and subsequently euthanized by decapitation. While under anesthesia, approximately 1 mL of cardiac blood was collected. Following decapitation, femur bones were collected and stored at −80°C until analysis. Blood samples were centrifuged at 3000 rpm for 15 min at 4°C to obtain serum samples. The bone and serum were stored at −80°C until further analysis.

### 2.2 Sample Processing and Spectroscopic Data Acquisition

For the Fourier‐transform infrared (FTIR) spectroscopy analysis, the soft tissues surrounding the bone specimens were removed. After removal of the surrounding soft tissues, femurs were processed as whole‐bone samples; bone marrow was not separately flushed prior to cryogenic grinding. The bones were cryogenically ground into a fine, homogeneous powder using a cooled mixer mill (Retsch MM 400, RETSCH Mill, Haan, Germany) operated at a frequency of 30 Hz for 6 min with liquid nitrogen.

The powdered samples were analyzed using ATR‐FTIR spectroscopy in the 4000–900 cm^−1^ wavenumber range. For each animal, three technical ATR‐FTIR spectra were acquired from the same powdered sample under identical conditions and averaged to obtain one representative spectrum per mouse (*n* = 6 biological replicates/group). Those represented spectra per mouse were used in advanced data analysis and statistical analysis.

### 2.3 FTIR Data Analysis

**FIGURE 1:**
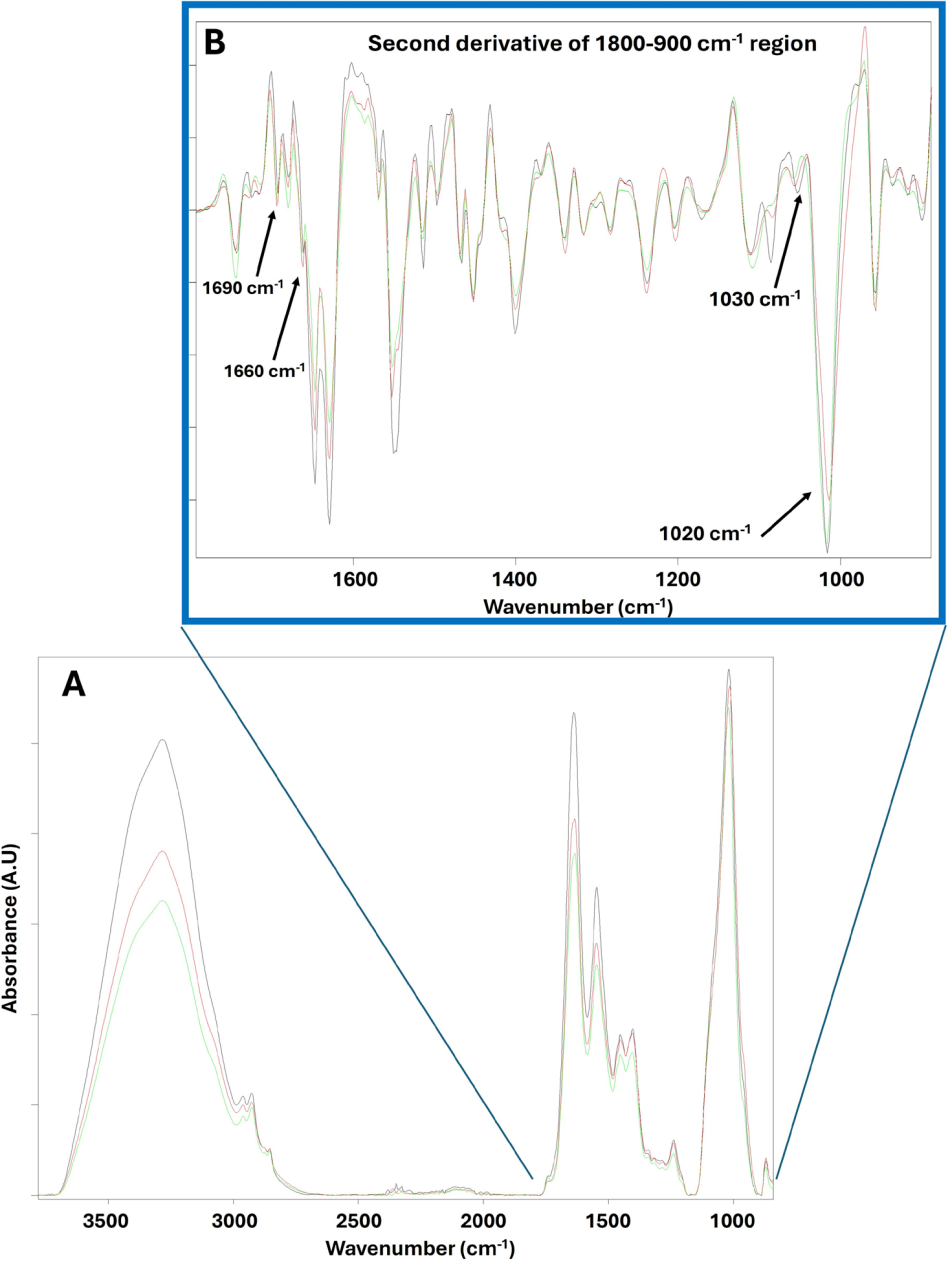
(A) Average ATR‐FTIR spectra obtained from healthy untreated control (C) (black in color), intubation gavage control (GC) (green), and alcohol‐treated (A) (red) mouse femoral bone in 3700–800 cm^−1^ wavenumber region; (B) second derivative spectra of Amide I and Phosphate symmetric stretching band.

Spectral analysis employed the Bruker OPUS Spectrum Analysis module (Version 8.5). From the spectra band shifting and intensity alterations, the molecular layer structural changes and organization variation metrics were collected. These metrics are indicators of molecular composition and structural organization variation (Algburi et al. 2022; Boskey and Mendelsohn 2005; Garip et al. 2013). Figure 1A presents an average ATR‐FTIR spectrum obtained from C, GC, and A mice femoral bone. Figure 1B represents the sub‐bands under Amide I and phosphate symmetric stretching bands. According to the literature, the assignment of the main bands in the spectrum shown in Figure 1A is given in Table 1. As demonstrated by the spectrum, the main vibrational characteristics of whole bone tissue are clearly identified, including bands due to lipid and protein components, as well as to mineral phases, particularly carbonate‐ and phosphate‐containing groups. The signal intensity and/or area under the bands originating from specific molecules in an FTIR spectrum are directly related to the concentration of corresponding functional groups in the system. Therefore, measuring the values and ratios of the area and intensity under the infrared bands provides information about the concentration of the molecule (Algburi et al. 2022; Benetti et al. 2024; Garip Ustaoglu et al. 2023).

**TABLE 1:** Assignment of the main bands of the mouse femur bone spectrum in 3000–900 cm^−1^ wavenumber region (Paschalis and Mabilleau 2025; Garip Ustaoglu et al. 2023; Algburi et al. 2022; Garip et al. 2013; Gourion‐Arsiquaud et al. 2009).

Band #	Wavenumber (cm^−1^)	Spectral band assignment
1	2956	Antisymmetric C‐H stretching vibrations of aliphatic CH_3_ groups: lipids and proteins
2	2923	Antisymmetric C‐H stretching vibrations of aliphatic CH_2_ groups: mainly lipids
3	2873	Symmetric C‐H stretching vibrations of aliphatic CH_3_ groups: mainly proteins
4	2854	Symmetric C‐H stretching vibrations of aliphatic CH_2_ groups: mainly lipids
5	1653	C = O stretching vibrations of amide groups: Amide I: protein
6	1545	N‐H bending vibrations accompanying C = O stretching vibrations in amide groups: Amide II: protein
7	1235	PO_2_^−^ antisymmetric stretching: phosphate molecules present in the structure of hydroxyapatite crystals
8	1080	PO_2_^−^ symmetric stretching: phosphate molecules present in the structure of hydroxyapatite crystals
9	890	Carbonate molecules present in the structure of hydroxyapatite crystals or free in the tissue

There are three main lipid‐related bands: CH_2_ symmetric (2854 cm^−1^), CH_2_ antisymmetric (2923 cm^−1^), and CH_3_ symmetric (2873 cm^−1^). The alterations in saturated lipid levels were analyzed by calculating the band area ratio of CH_2_ symmetric/CH_2_ symmetric + CH_2_ antisymmetric; the aliphatic chain length was obtained by dividing the area of the CH_2_ antisymmetric band by the area of the CH_3_ symmetric band (Garip Ustaoglu et al. 2023). The alteration in the concentration of proteins was calculated using the two main protein bands in the spectrum, Amide I (1653 cm^−1^) and Amide II (1545 cm^−1^). Total protein content was calculated using the ratio of Amid I/Amid I + II band areas (Algburi et al. 2022). Moreover, Amide I/Amide II band area ratio was also measured.

There are four infrared (IR) spectroscopic ratio parameters that are regularly used to assess the tissue quality of bone. These ratio parameters are namely mineral‐to‐matrix, carbonate‐to‐phosphate, collagen cross‐link, and crystallinity. The mineral‐to‐matrix ratio that gives information about bone mineral density indicates the proportion of minerals in relation to the organic matrix. It is calculated by dividing the areas of the symmetric phosphate band (1080 cm^−1^) to the Amide I band (Benetti et al. 2024). The carbonate‐to‐phosphate ratio that reflects the extent to which carbonate has substituted for phosphate in hydroxyapatite was calculated using the band area of the carbonate band (890 cm^−1^) to the phosphate band (Bayarı et al. 2020; Garip Ustaoglu et al. 2018). There are sub‐bands under Amide I and phosphate symmetric stretching bands which cannot be seen from the first derivative spectra but are visible in second derivative spectra as seen in Figure 1B. Thus, these sub‐band‐derived ratios (collagen cross‐link and crystallinity ratios) were obtained from second derivative spectra using the analysis workflow implemented in the Bruker OPUS software. The collagen cross‐link ratio is derived from the Amide I protein band by taking the intensity ratio of the sub‐bands at 1660 and 1690 cm^−1^ (Garip et al. 2013; Lodoso‐Torrecilla et al. 2024). Finally, crystallinity is assessed from the phosphate ν₁, ν₃ region as the intensity ratio of the 1030–1020 cm^−1^ sub‐bands (Garip Ustaoglu et al. 2018; Taylor and Donnelly 2020). The degree of structural order within a mineral is referred to as mineral crystallinity and is commonly assessed through X‐ray diffraction. It shows that the crystal size in addition to how well the atoms are arranged.

### 2.4 Enzyme‐Linked Immunosorbent Assay (ELISA) Analysis

The measurement of serum parathyroid hormone (SunRed 201‐11‐0334), C‐telopeptide (Nepenthe NE06C222103), 25‐hydroxyvitamin D (Nepenthe NE06C789026), osteocalcin (Nepenthe NE06S239103), and calcitonin (SunRed 201‐11‐0347) levels was measured according to the manufacturer's protocol performing enzyme‐linked immunosorbent assay on 96‐well plates and reading the response on a microplate reader (Biochrom EZ Read 400). Each serum sample was assayed in duplicate, and the mean of the two technical measurements was used for statistical analysis. This method allows the measurement of markers indicative of calcium homeostasis and bone turnover. The measured data were used to assess the effects of alcohol exposure and procedural stress with respect to the markers of bone formation and bone resorption. Biochemical analyses were interpreted by considering both C versus GC and GC versus A comparisons. Those biochemical effects were then correlated with changes in bone molecular structure, which were measured and analyzed by ATR‐FTIR spectroscopy.

### 2.5 Statistical Evaluation

Data distributions were first assessed using the Kolmogorov–Smirnov test. Variables meeting normality assumptions were analyzed by one‐way ANOVA followed by Tukey's multiple‐comparisons test. All analyses were conducted in GraphPad Prism (v6.05; GraphPad Software, San Diego, CA, USA). Data are presented as mean ± SD. The statistical significance was defined as *p* ≤ 0.05, with the significance levels indicated as *,^#^ *p* ≤ 0.05, **,^##^ *p* ≤ 0.01, and ***,^###^ *p* ≤ 0.001. In figures and tables, “*” indicates comparison versus C, and “#” indicates comparison versus GC.

## 3 Results

In the current study, the long‐term effects of early postnatal alcohol exposure and gavage‐related stress on bone molecular structure and bone‐related biochemical markers were investigated using ATR‐FTIR spectroscopy and ELISA assays.

### 3.1 Growth and Body Weight

**FIGURE 2:**
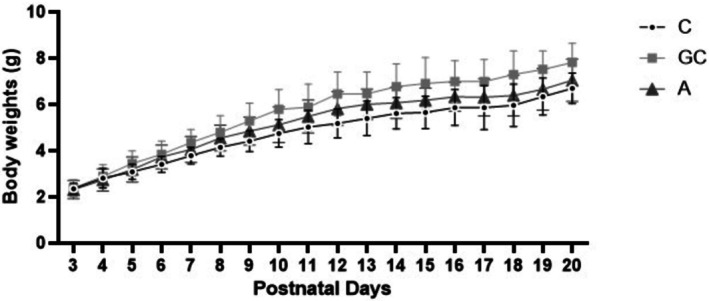
Daily body weight alterations during the treatment period (PND3–PND20) for untreated control (C), intubation gavage control (GC), and alcohol‐treated (A) groups.

The growth curves for the control (C), gavage control (GC), and alcohol‐treated (A) groups from Postnatal Day (PND) 3 to PND 20 are presented in Figure 2. Statistical analysis of body weight revealed no significant differences between the three experimental groups (*p* = 0.109). These findings suggest that, while postnatal alcohol exposure and gavage‐related stress significantly altered the molecular composition of the bone, they did not result in long‐term changes to total body mass or generalized growth retardation.

### 3.2 ATR‐FTIR Spectroscopic Results

**FIGURE 3:**
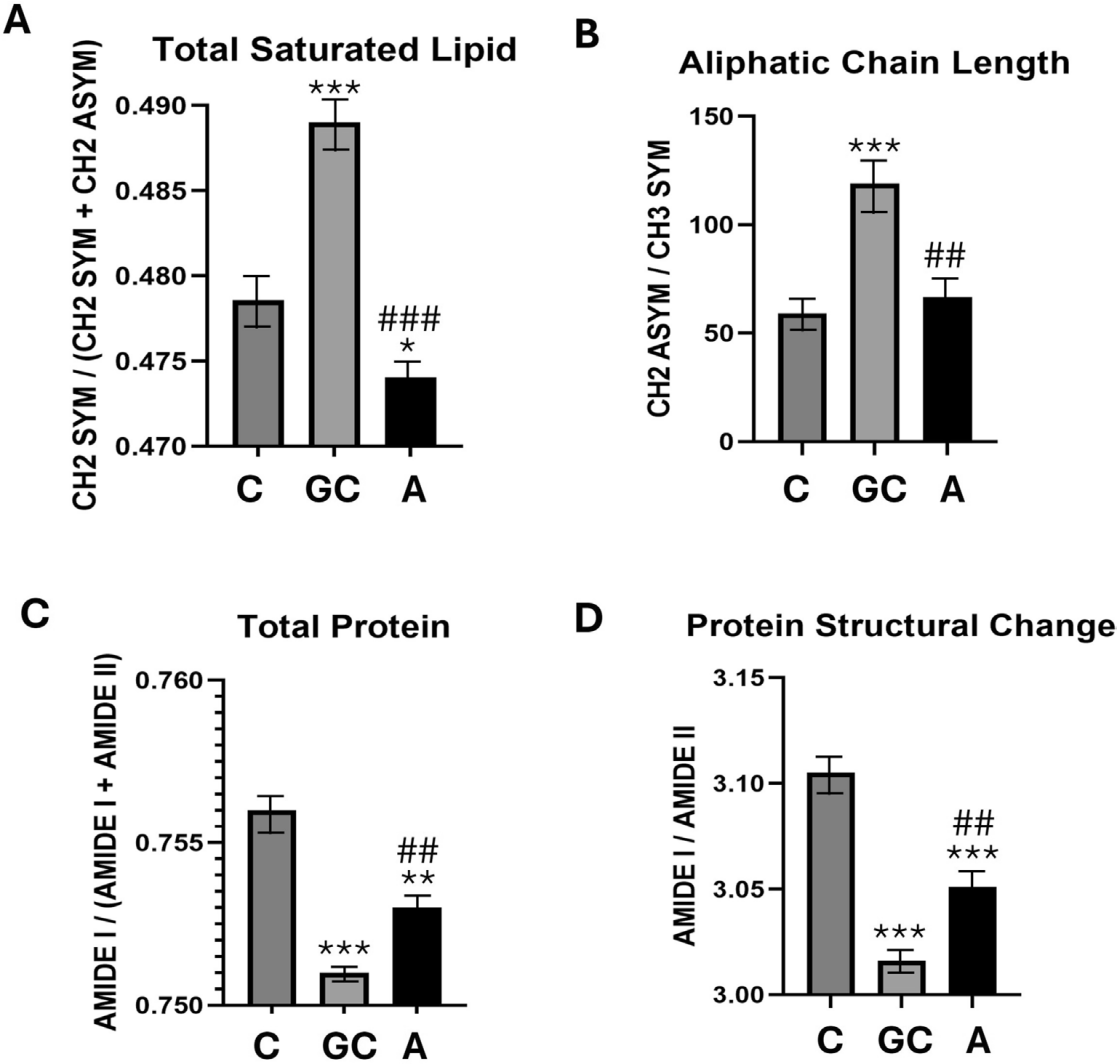
Variations in (A) total saturated lipid, (B) aliphatic chain length, (C) total protein, and (D) protein structural change spectral parameters for untreated control (C), intubation gavage control (GC), and alcohol‐treated (A) groups. *,^#^ *p* ≤ 0.05, **,^##^ *p* ≤ 0.01, and ***,^###^ *p* ≤ 0.001. “*” indicates comparison versus C, whereas “#” indicates comparison versus GC.

**FIGURE 4:**
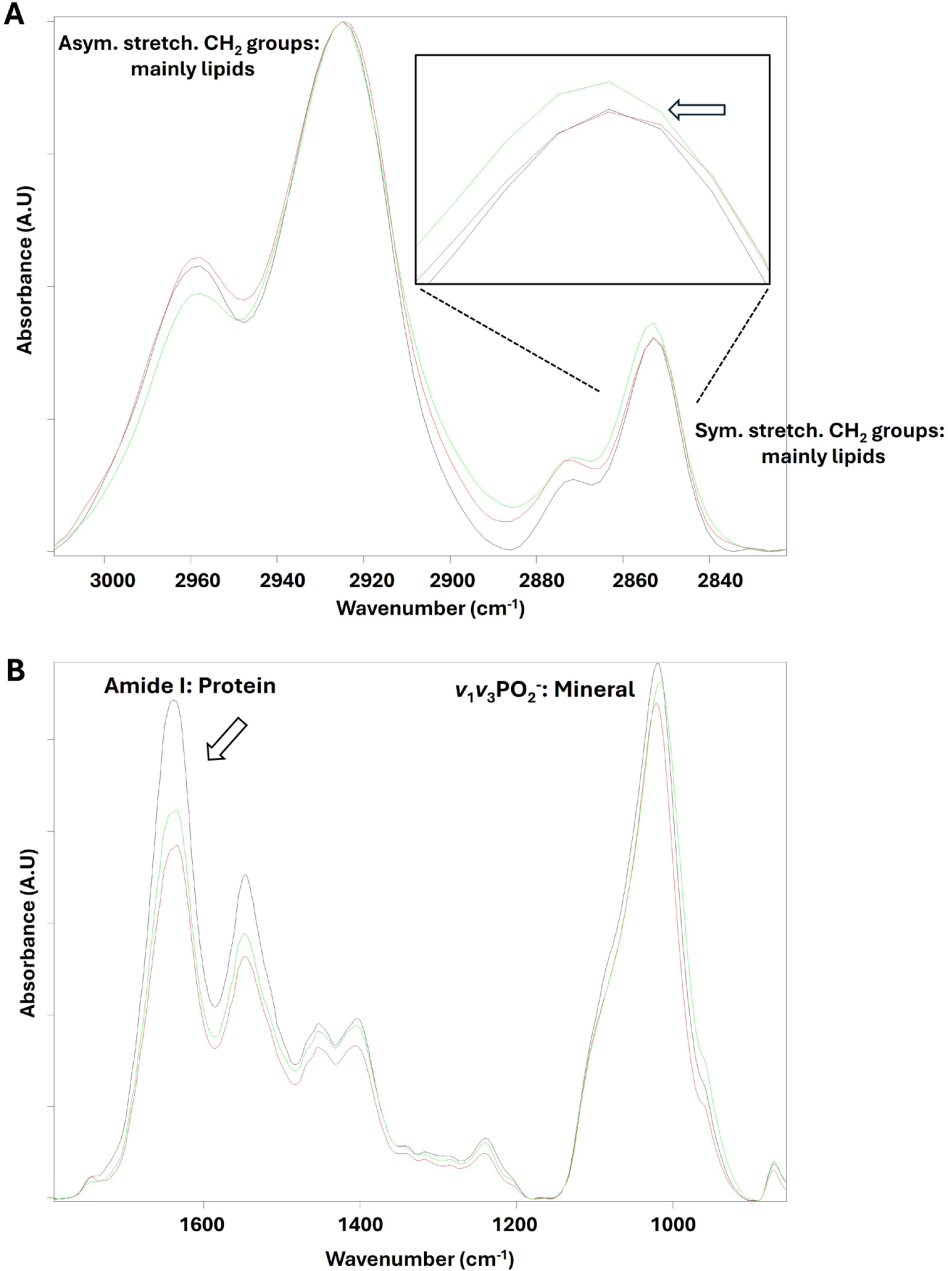
Average spectra of untreated control (C) (black in color), intubation gavage control (GC) (green), and alcohol‐treated (A) (red) groups in the wavenumber range of (A) 3000–2820 cm^−1^ and (B) 1800–800 cm^−1^. The spectra were normalized according to CH_2_ antisymmetric stretching band at 2923 cm^−1^.

Figure 3 shows spectral alterations in the ratios of areas under the infrared bands obtained from the C, GC, and A groups. The comparison of the C group with the A group demonstrates the effects of alcohol on bone tissue, while the comparison of the GC group with the C group demonstrates the alcohol‐independent effects of stress due to gavage on bone tissue. In addition, Figure 4 demonstrates the average spectra of those groups in the range of (A) 3000–2820 cm^−1^ and (B) 1800–800 cm^−1^.

Exposure to alcohol caused a significant reduction in the saturated lipid ratio in bone tissue, whereas physiological stress resulted in an increase in this ratio (Figure 3A). This alteration can also be clearly seen from CH_2_ symmetric band at 2854 cm^−1^ in Figure 4A. Moreover, the aliphatic chain length significantly increased in the GC group when compared to the C group, while this ratio significantly decreased in the A group compared to GC group (Figure 3B).

As seen from Figure 3C, total protein content significantly decreased in both the GC and A groups. This alteration can also be clearly observed in the Amide I band at 1653 cm^−1^ in Figure 4B. Furthermore, the Amide I/Amide II band area ratio was also significantly reduced in both the GC and A groups (Figure 3D) as similar to the total protein content result.

The variations in the spectroscopic ratio parameters (mineral‐to‐matrix, carbonate‐to‐phosphate, collagen cross‐link, and crystallinity) indicating bone quality for the studied groups are given in Table 2.

**TABLE 2:** Variations in bone quality ratio parameters for untreated control (C), intubation gavage control (GC) and alcohol‐treated (A) groups. *,^#^ *p* ≤ 0.05, **,^##^ *p* ≤ 0.01, and ***,^###^ *p* ≤ 0.001. “*” indicates comparison versus C, whereas “#” indicates comparison versus GC.

	Control (C)	Gavage control (GC)	Alcohol‐treated (A)
Bone mineral content ratio (PO_2_^−^ symmetric stretching/Amid I)	0.0516 ± 0.008	0.0459 ± 0.004 **	0.0505 ± 0.002 *; #
Relative carbonate content ratio (*ν2* carbonate/PO_2_^−^ symmetric stretching)	0.5809 ± 0.125	0.5914 ± 0.170	0.4860 ± 0.084 *; #
Collagen cross‐link ratio (1660 cm^−1^/1690 cm^−1^)	0.3603 ± 0.020	0.2802 ± 0.085 ***	0.3210 ± 0.053 *; #
Crystallinity ratio (1030 cm^−1^/1020 cm^−1^)	0.1302 ± 0.015	0.1736 ± 0.021 **	0.1583 ± 0.028 *; #

As shown in Table 2, a decrease in bone mineral content was observed in both the GC and A groups compared to the C group implying that this alteration was not specific to alcohol exposure. This decrease can also be observed in the main mineral band *v*_1_ *v*_3_ PO_2_^−^ at 1080 cm^−1^ (Figure 4B). The relative carbonate content ratio was significantly lower in the A group than in both the C and GC groups (Table 2), suggesting an alcohol‐related effect on mineral composition beyond the gavage‐related stress. Therefore, these parameters are informative in the amount of minerals, the mechanical properties of minerals, as well as the degree of carbonate substitution within the apatite lattice (Garip et al. 2013). As seen from Table 2, collagen cross‐link ratio was reduced in both treated groups relative to the C group, while the A group showed partial attenuation relative to the GC group, indicating that this parameter was strongly influenced by gavage‐related stress. Moreover, the crystallinity parameter increased in both the GC and A groups relative to the C group, with the A group not exceeding the GC group, implying that increased crystallinity was primarily associated with the gavage‐related stress rather than a uniquely deleterious alcohol effect (Table 2).

Together, these four IR outcomes provide a concise, complementary profile of bone matrix composition and organization.

### 3.3 ELISA Study Results

**FIGURE 5:**
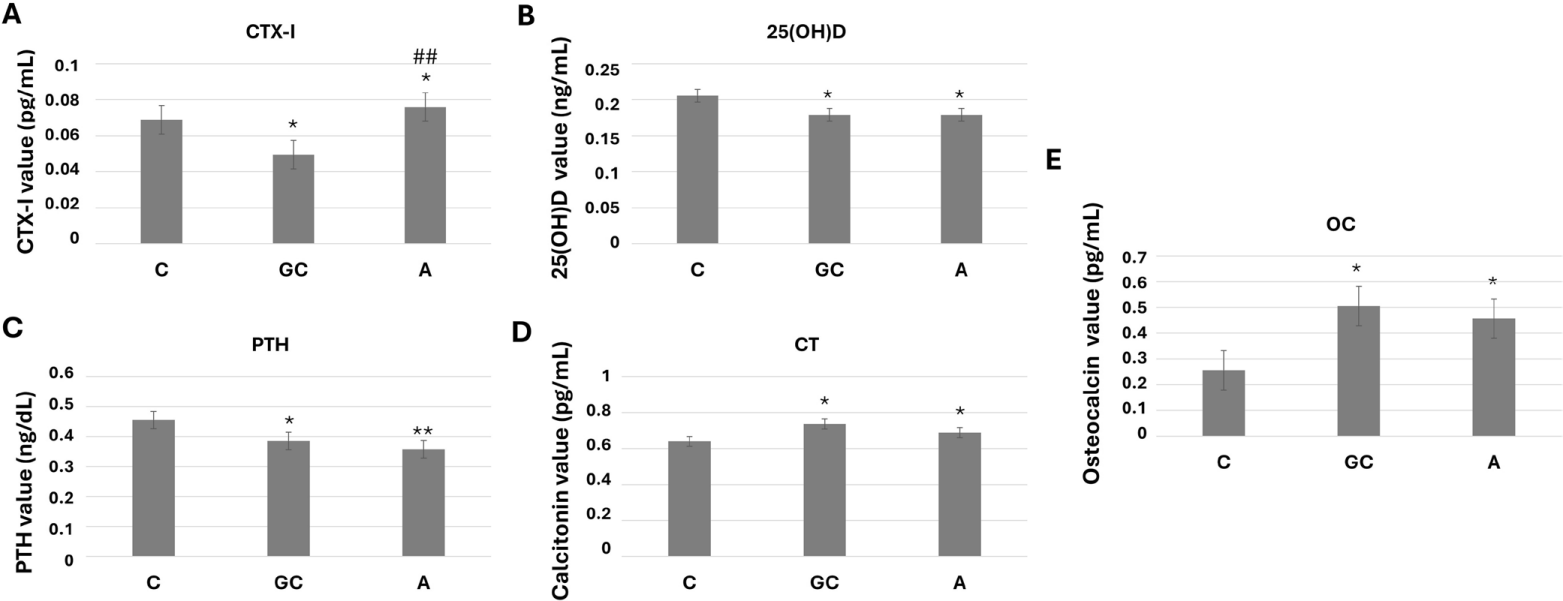
Variations in serum values of (A) C‐telopeptide (CTX‐I), (B) 25‐hydroxyvitamin D [25(OH)D], (C) parathyroid hormone (PTH), (D) calcitonin (CT), and (E) osteocalcin (OC) for untreated control (C), intubation gavage control (GC), and alcohol‐treated (A) groups. *,^#^ *p* ≤ 0.05, **,^##^ *p* ≤ 0.01, and ***,^###^ *p* ≤ 0.001. “*” indicates comparison versus C, whereas “#” indicates comparison versus GC.

Research using ELISA tests was conducted to assess the factors that are important for understanding bone calcium metabolism and indicators of bone turnover. CTX‐I was elevated in the A group relative to both controls and represented the most consistent alcohol‐associated biochemical change (Figure 5A). In contrast, 25(OH)D, PTH, osteocalcin, and calcitonin changes were observed in both the GC and A groups relative to the C group, and there was no significant change in those parameters in the A group compared to the GC group, indicating that these endocrine alterations were not specific to alcohol exposure alone.

As illustrated in Figure 5B, both the A and GC groups were significantly lower in the serum 25(OH)D concentrations than the C group, while there is no significant alteration between the GC and A groups. Although, compared to the C group, both the GC and A groups had significantly lower levels of PTH, slight changes in PTH levels were found between the A and GC groups (Figure 5C). As seen in Figure 5D, as a PTH suppressant, calcitonin levels were considerably higher in both gavage groups (A and GC) compared to the C group, while there were no significant changes between the A and GC groups. Moreover, osteocalcin levels showed a significant increase in both the GC and A groups compared with C (Figure 5E). In contrast, group A did not differ significantly from the GC group. These findings indicate that the observed increase in osteocalcin was associated with the gavage‐related stress rather than an alcohol‐related effect.

Therefore, the hormonal findings mostly showed stress‐associated changes rather than uniquely alcohol‐driven effects.

## 4 Discussion

The current study was designed to determine the effects of postnatal alcohol exposure on bone molecular composition. In addition, stress‐related alterations were also noted due to intragastric intubation by gavage. In this context, the comparison between the control and alcohol‐treated groups provides important insight, indicating that the observed differences in bone‐related outcomes are more likely attributable to gavage‐related stress than to alcohol exposure itself. Many of the ATR‐FTIR spectroscopic alterations observed in alcohol‐treated group, including reduced total protein‐related indices, lower collagen cross‐link ratio, reduced mineral‐to‐matrix ratio, and increased crystallinity, were already present in the gavage control group. Likewise, several endocrine changes were shared by the gavage control and alcohol‐treated groups when compared with untreated controls. Taken together, these findings suggest that prolonged neonatal gavage was not a neutral intervention in this model and may have contributed substantially to the long‐term skeletal phenotype.

ATR‐FTIR spectroscopy is widely used to characterize bone tissue quality, providing information complimentary to bone mineral density by quantifying parameters such as mineral‐to‐matrix, carbonate‐to‐phosphate, collagen cross‐linking, and crystallinity. Spectroscopic measurements of the mineral‐to‐matrix, carbonate‐to‐phosphate, collagen cross‐link, and crystallinity indices have been shown to correlate with in vivo and clinical studies of the bones with respect to their mechanical properties, fracture risk, and disease (Paschalis and Mabilleau 2025).

Within the spectroscopic dataset, the overall pattern points to a marked influence of gavage‐related stress on both the organic and mineral components of bone. The decreases in total protein‐related indices and collagen cross‐link ratio in the gavage control group may reflect altered organization or maturation of the bone matrix (Zoehrer et al. 2008), while the lower mineral‐to‐matrix ratio and higher crystallinity suggest that the mineral phase was also affected. Such changes are commonly linked to a reduction in bending stiffness and an increase in the likelihood of fracture (Wehrle‐Martinez et al. 2023). Higher crystals consequently result in higher structural organization and larger mineral component crystals. However, this increase has been correlated with more fragile bone tissue and diminished fracture resistance (Paschalis et al. 2011). Because similar changes were observed in the alcohol‐treated group relative to the untreated controls, these differences should not be interpreted as alcohol‐specific on their own. Rather, the data indicate that a considerable part of the long‐term variation in bone molecular composition was observed by both gavaged groups. This result argues against attributing the full pattern of matrix changes specifically to alcohol.

When the alcohol‐treated group was compared directly with the gavage control group, the pattern was more selective. Among the FTIR‐derived parameters, the clearest additional difference was the lower relative carbonate‐to‐phosphate ratio in the alcohol‐treated group. This finding may indicate an alcohol‐associated modification of mineral composition beyond the effect of gavage‐related stress alone. It has been reported that altered carbonate to phosphate ratios are typical of osteoporotic or fragile bone (Benetti et al. 2024). However, this interpretation should remain cautious, because FTIR provides relative compositional information and, in the absence of complementary structural or mechanical analyses, it cannot determine the functional consequences of this difference.

This more selective pattern is also in line with previous experimental evidence suggesting that alcohol may exert direct effects on bone‐forming cells. In vitro studies by Pedersen et al. (2025) demonstrated that elevated acetate levels, a hepatic ethanol metabolite, suppressed the growth and proliferation of mouse preosteoblasts and osteoblasts (Pedersen et al. 2025). Alcohol exposure has also been reported to affect osteoblast‐ and osteoclast‐related gene expression and to disrupt growth plate morphology. Although the present study does not allow direct mechanistic conclusions, these observations may provide a biological context for the additional alcohol‐associated differences observed relative to the gavage control group.

The lipid‐related findings also support the view that gavage and alcohol were associated with partly overlapping but not identical effects. Saturated lipid ratio increased in the gavage control group, whereas it decreased in the alcohol‐treated group relative to untreated controls, while aliphatic chain length significantly increased in just the GC group. These observations suggest that gavage‐related stress and alcohol may influence membrane‐associated spectral features in different ways, but the biological significance of these shifts remains uncertain in the absence of cell‐specific or region‐specific analyses. Recent studies have established a link between bone loss and disorders related to alteration of lipid content and/or structure including atherosclerotic vascular diseases and dyslipidemia. The important effect of lipid metabolism on skeletal health and the increased risk of fracture were also highlighted (Kim et al. 2021). Aleskos and colleagues (2020) reported the relationship between dyslipidemia and bone fragility with a decrease in BMD. Moreover, some specific fatty acids (FAs) including conjugated linoleic acid and omega‐3 FA were shown to play a role in the bone remodeling process (Luo et al. 2025). In the current study, since the FTIR measurements were performed on whole‐bone powders, a limited contribution from marrow‐derived lipids and proteins also cannot be excluded and should be considered when interpreting these signals.

Both gavage control and alcohol‐treated groups demonstrated reduced total protein content and Amide I/Amide II ratio variations, suggesting a structural modification and reduction in the bone protein matrix. Previous studies have reported similar FTIR results in osteoporotic or fractured bone, including reduced Amide I contribution, decrease in collagen maturity, and increased crystallinity, although densitometry changes are insignificant (Boskey and Camacho 2007; Gourion‐Arsiquaud et al. 2009). The changes in both lipids and proteins indicate that stress and alcohol affect the organic matrix that regulates mineral deposition and determines mechanical properties.

The alterations in bone quality detected spectroscopically have been corroborated by the findings in the ELISA analyses. The biochemical findings similarly indicate that most endocrine alterations were not unique to alcohol exposure. Serum 25(OH)D, PTH, osteocalcin, and calcitonin differed from untreated controls in both gavaged groups, whereas no clear difference was observed between the alcohol‐treated and gavage control groups. Overall, these results suggest that much of the hormonal pattern was associated with the gavage procedure itself rather than with an additional alcohol‐specific effect. Because total or ionized serum calcium was not measured, these hormonal data should be interpreted as alterations in calcium‐regulating hormones rather than as direct evidence of disturbed systemic calcium homeostasis.

Among the serum markers examined, CTX‐I represented the most consistent additional difference in the alcohol‐treated group relative to the gavage control group. This finding may indicate an alcohol‐associated shift in resorption‐related turnover beyond the effect of the gavage alone. However, this interpretation should remain cautious. In the absence of bone histology, histomorphometry, microCT, or mechanical testing, the present data do not allow firm conclusions regarding osteoclast activity, high‐turnover bone loss, or overall bone fragility. Accordingly, CTX‐I is better interpreted here as an alcohol‐associated biochemical difference that may warrant further investigation, rather than as definitive evidence of a specific cellular mechanism.

The concurrent rise in calcitonin levels in both the gavage control and alcohol‐treated groups may reflect a compensatory endocrine response. Calcitonin is known to inhibit osteoclast activity and thereby reduce bone resorption, and increased calcitonin levels have been reported in association with alcohol exposure, although the literature is not fully consistent (Babić Leko et al. 2021). In the present dataset, the absence of a corresponding increase in CTX‐I in the gavage control group may be compatible with the possibility that counter‐regulatory pathways contributed to limiting resorption‐related changes in animals exposed only to the gavage‐related stress. Nevertheless, this interpretation remains speculative and cannot be confirmed from the current data alone.

Likewise, lower 25(OH)D and PTH levels in both the gavage control and alcohol‐treated groups may be discussed considering previous reports linking alcohol use with vitamin D deficiency and altered parathyroid hormone regulation (Laitinen and Välimäki 1991; Neupane et al. 2013). However, because similar changes were also present in the gavage control group, the current findings do not support interpreting these hormonal alterations as alcohol specific. Rather, they suggest that prolonged gavage, with or without alcohol, may have influenced endocrine parameters related to mineral regulation. Since serum calcium concentrations were not available, these observations should not be taken as evidence of confirmed disruption in calcium homeostasis.

The hormone osteocalcin is a marker of bone formation and is part of the energy metabolism and acute stress response pathways. During psychological stress, serum levels of osteocalcin may rise rapidly in rodents (Berger and Karsenty 2022). Therefore, the alteration of osteocalcin in the gavage control animals possibly reflects an adaptation to stress in bone turnover that does not result in observable bone resorption. Previous studies reported that osteocalcin often does not correlate with resorption markers (Martiniakova et al. 2024; Schini et al. 2023). The absence of a significant osteocalcin alteration in the alcohol‐treated group compared to the gavage control, coupled with high levels of resorption markers and spectral evidence of diminished mineral content, is consistent with this reported uncoupling.

The absence of significant differences in body weight across the study period is also relevant for interpretation. Since neither gavage nor alcohol exposure produced lasting differences in body mass, the observed molecular and biochemical changes are less likely to be explained simply by persistent generalized growth suppression. Nevertheless, skeletal maturation was not assessed directly by bone length, bone mass, microarchitecture, or biomechanical testing. Therefore, the present findings should be interpreted primarily as evidence of long‐term compositional and hormonal associations rather than a complete characterization of skeletal outcome.

Several limitations should be considered. First, ATR‐FTIR spectroscopy yields relative rather than absolute compositional measures and was applied here to whole‐bone powder, which does not resolve cortical versus trabecular compartments or newly formed versus older bone tissue. Second, bone marrow was not removed prior to analysis, so a limited contribution from marrow‐derived components cannot be excluded. Third, no direct marker of procedural stress, such as corticosterone, was measured; thus, the involvement of stress is inferred from the gavage control group versus untreated control group comparison rather than demonstrated directly. Fourth, serum calcium concentrations were not available, so the study cannot establish disruption of systemic calcium homeostasis. Finally, the lack of mechanical testing and imaging‐based bone phenotyping limits conclusions about bone strength, structure, and fracture susceptibility.

In summary, the present findings indicate that prolonged neonatal gavage‐related stress was associated with substantial long‐term changes in bone molecular composition and in several circulating bone‐related hormonal markers. Against this background, the additional effects associated with alcohol exposure appeared more limited, with the most consistent differences relative to gavage controls observed for CTX‐I and relative carbonate content. These results underscore the importance of separating gavage‐related effects from alcohol‐associated effects in developmental models based on repeated neonatal gavage and support a cautious interpretation of endocrine and matrix findings when direct measurements of calcium status, bone structure, and bone mechanics are not available. This emphasizes the necessity of taking stress levels into consideration during the interpretation of bone study outcomes in rodent models, thereby supporting the expanding demand for the adoption of less stressful oral dosing methodologies wherever possible.

## 5 Conclusion

Prolonged neonatal gavage‐related stress was associated with substantial long‐term changes in bone molecular composition and in several circulating bone‐related hormonal markers in female mice. Many of the alterations observed in the alcohol‐treated group were also present in the gavage control group, indicating that the gavage‐related stress itself contributed importantly to the overall phenotype. Relative to the gavage controls, postnatal alcohol exposure was associated with additional selective differences, most notably in CTX‐I and relative carbonate content. Because serum calcium, bone imaging, and mechanical properties were not assessed, these findings should be interpreted cautiously and not as direct evidence of disturbed systemic calcium homeostasis or definitive impairment of bone strength.

## Author Contributions

**Ilknur Dursun:** writing – review and editing, writing – original draft, validation, methodology, investigation, data curation, conceptualization. **Birsen Elibol:** writing – original draft, writing – review and editing, conceptualization. **Sebnem Garip Ustaoglu:** writing – review and editing, writing – original draft, validation, methodology, investigation, funding acquisition, formal analysis, data curation, conceptualization.

## Funding

This work is part of a research project funded by Altinbas University, Research Council, (number: PB2022‐TIP‐3).

## Disclosure

The authors declared no generative AI tools were used in the preparation of this manuscript.

## Conflicts of Interest

The authors declare no conflicts of interest.

## Data Availability

The data that support the findings of this study are available from the corresponding author upon reasonable request.
